# Advances in Medical Nutrition Therapy: Parenteral Nutrition

**DOI:** 10.3390/nu12030717

**Published:** 2020-03-08

**Authors:** Moran Hellerman Itzhaki, Pierre Singer

**Affiliations:** Department of General Intensive Care, Institute for Nutrition Research, Rabin Medical Center, Beilinson Hospital, Petah Tikva 49100, Israel; MORANHE@clalit.org.il

**Keywords:** parenteral nutrition, lipid emulsion, glucose control, Indirect calorimetry

## Abstract

Parenteral nutrition has evolved tremendously, with parenteral formulas now safer and more accessible than ever. “All-in-one” admixtures are now available, which simplify parenteral nutrition usage and decrease line infection rates alongside other methods of infectious control. Recently published data on the benefits of parenteral nutrition versus enteral nutrition together with the widespread use of indirect calorimetry solve many safety issues that have emerged over the years. All these advances, alongside a better understanding of glycemic control and lipid and protein formulation improvements, make parenteral nutrition a safe alternative to enteral nutrition.

## 1. Introduction

When providing nutrition support to a patient, the oral route is the preferred option. Many acute and chronic medical conditions, such as dysphagia or reduced levels of consciousness, do not allow the use of oral nutrition. In these cases, enteral nutrition should be given to support the patient’s nutritional needs. Parenteral nutrition provides intravenous nutrition for patients who are unable or cannot tolerate enteral nutrition, such as patients with intestinal failure, paralytic ileus, bowel ischemia, etc. It has been more than half a century since parenteral nutrition was first introduced. In the past, primary formulas were rich in glucose, since lipid emulsions were not available, and proteins were mainly large and not properly utilized. Over time, with advancements in technology, significant changes and improvements were made in order to make the formulas more physiological and accessible with fewer significant side effects. Furthermore, better understanding of patients’ needs allowed parenteral nutrition solutions to be individualized according to the patient or clinical condition. This review summarizes the latest changes made in parenteral nutrition.

## 2. Advances in Pharmaceutical Preparation: “All-in-One” Admixtures

**Figure 1:**
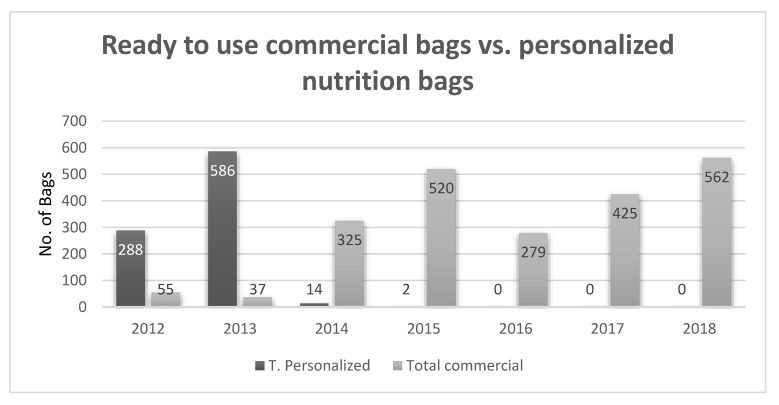
Personalized compound bags vs. ready-to-use, electrolyte-free commercial bags throughout the years (internal data).

Historically, parenteral nutrition was administered in separate bottles containing a carbohydrate solution, an amino acid hydrolysate, and a lipid emulsion together with vitamins and trace element vials. Over the last few decades, all-in-one (three-in-one) admixture (AIO) systems for parenteral nutrition have become available [1,2]. The use of these systems prevents component manipulation, thereby reducing the probability of contamination. This method requires only one intravenous access, lowering the risk of infection. A recent literature review showed that the use of all-in-one admixtures had significant advantages regarding rates of bloodstream infections and therefore length of stay [3]; a summary of these studies is shown in Table 1. AIO systems provide simpler prescriptions, save time, and reduce workload and costs [4]. In a paper published by *Pichard* et al., a significant reduction in preparation time was shown throughout all levels of manpower, including the physician’s prescription, the nurse’s administration and preparation, and the pharmacist’s compounding total parenteral nutrition (TPN). In total, 25 min was spent using the separated bottle system compared to 11 min for the AIO system [5]. There are two types of AIO systems, namely, personalized compound bags which are prepared in hospitals or industry pharmacies, and “ready-to-use” commercial bags. Personalized compound bags were designed to meet the nutritional needs of the patient in relation to specific clinical conditions. When using a “ready-to-use” commercial bag, patient-specific nutritional requirements must be considered, therefore, despite the advances in AIO commercial bags, many clinical centers worldwide still prefer personalized compound bags [6]. It is important to note that not all centers have a skilled pharmacist for compounding TPN, a problem which can be eliminated by using AIO commercial bags. A recently conducted observational study in our center reported a dramatic decrease in the use of personalized compound bags since 2014 [7] (see Figure 1). This decrease was possible when using electrolyte-free formulas, as well as a large variety of volume bags (1, 1.5, 2.0, and 2.5 L). This allowed the use of a partial bag if desired and the addition of electrolytes depending on the patient’s recent lab results. All additions to commercial bags, including vitamins and trace elements, are performed according to the manufacturer’s recommendations, thereby maintaining the stability of the formula.

## 3. Consideration for Support of Parenteral Nutrition

### 3.1. Enteral Versus Parenteral Nutrition

While the importance of nutritional support is well documented, the preferred route for nutritional delivery is still debatable. Both forms of nutrition have advantages and disadvantages. Parenteral nutrition (PN) has been associated with more infectious complications according to multiple meta-analyses [8,9], however, caloric targets are more easily reached using this method [10]. Alternatively, enteral nutrition (EN) preserves gastric function due to it being a more physiological route [11], but is associated with higher rates of gastric and intestinal intolerance [12], such as vomiting, reflux, aspiration, and even ischemic bowel syndrome. In 2011, the EPaNIC trial showed reduced rates of infection when delaying parenteral nutrition initiation [13]. Data gathered from Nutrition day (2016) by ESPEN showed a dramatic decrease in the use of parenteral nutrition and a delay in worldwide parenteral nutrition initiation in 2011, which was around the time of the EPaNIC trial publication. In recent years, the use of parenteral nutrition has progressively increased and the early use of parenteral nutrition is becoming common once again [14]. Results from the CALORIES trial [15] were published in 2014, which was a randomized controlled trail (RCT) comparing EN to PN in critically ill patients, in which nutritional support was initiated within 36 h of admission. The data showed no difference in the 30-day mortality rates. It is important to note that most of the patients did not reach their caloric target (25 kcal/kg/day), and their caloric intake was around 20 kcal/kg/day. A recently published randomized control trial, NUTRIREA-2, investigated the effect of EN versus PN in critically ill patients with shock who required invasive mechanical ventilation and vasopressor support. The 28-day mortality rates did not differ between the two groups and there was no significant different in the rate of infection. However, the results did show a significantly higher risk of gut ischemia in severely ill patients receiving enteral nutrition [16]. In the 2017 European Society of Intensive Medicine (ESICM) clinical practice guidelines, early EN is preferred over early PN. In their meta-analysis, EN usage did not show a mortality benefit compared to PN, but the risk of infection was reduced [17]. In the recently published guidelines on clinical nutrition in intensive care by the European Society of Clinical Nutrition and Metabolism (ESPEN), the use of EN over PN is recommended in patients with intact gastrointestinal tracts. However, parenteral nutrition is clearly indicated if enteral nutrition or caloric targets are not feasible. In these cases, PN should be prescribed mainly if the patient is severely malnourished [18]. All of these guidelines are unanimous in recommending PN when EN is not possible or is insufficient. The timing of nutritional support is another key question, but studies show conflicting results. In a large multicenter RCT by *Casaer* et al. [13], early supplemental parenteral nutrition (started after 48 h of admission) was compared to late supplemental parenteral nutrition (after eight days of hospitalization) in critically ill patients. They found that patients in the late initiation group had lower rates of infection, a higher chance of earlier intensive care unit (ICU) and hospital discharge, and a smaller chance of requiring prolonged mechanical ventilation and renal replacement therapy [13]. Doig et al. examined the effects of early parenteral nutrition in critically ill patients when enteral nutrition was contraindicated. Comparing PN in the first 24 h of admission to standard care did not show any statistically significant differences in mortality, quality of life, or infection [19]. In the early phases of a disease, increased endogenous energy substrates are released, which continues despite energy administration and can result in overfeeding [20]. As mentioned above, both studies involved starting nutrition support at a very early stage of the illness, which may explain some of the results. *Heidegger* et al. showed that reaching 100% of the patient’s energy requirements between day four and day eight of admission using supplemental parenteral nutrition reduced the rate of nosocomial infection. All of the patients in the study underwent indirect calorimetry measurements and supplemental PN was given in order to reach target energy expenditure. The rate of nosocomial infection was significantly lower in the PN group, with a hazard ratio of 0.65 [21]. There is a consensus regarding the safety of parenteral nutrition when it must be administered to patients intolerant to enteral nutrition. Supplemental administration of parenteral nutrition in patients tolerating partially enteral nutrition is still debatable, especially regarding the PN start date, which can be anywhere from day three to day seven.

### 3.2. The use of Indirect Calorimetry

Indirect calorimetry (IC) has been long proven to be the gold standard for resting energy expenditure assessment [18,22,23,24], however, technical difficulties have limited its use. Additionally, certain clinical situations, such as mechanical ventilation with an FIO2 above 0.7, the use of thoracic drainage, and the use of nitric oxide or helium, make IC measurements unpredictable.

**Figure 2:**
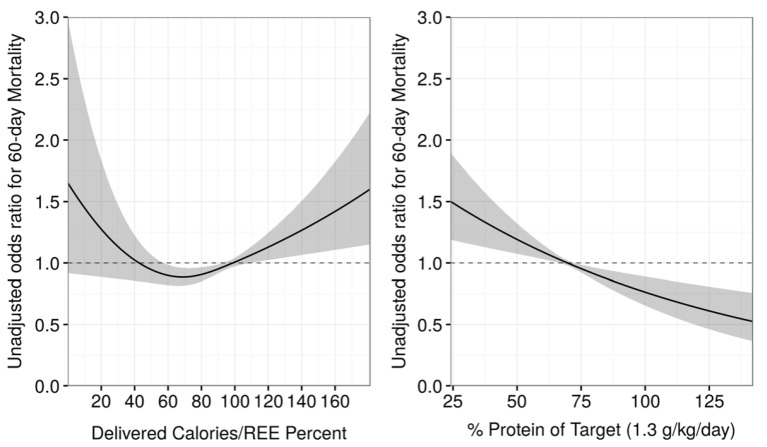
Association of administered caloric/resting energy expenditure (Adcal/REE) percent according to 60-day mortality (**left**) and protein intake by daily requirement (1.3g/kg/day), with 60-day mortality (**right**) presented using the odds ratio. REE: Resting energy expenditure. Reprinted from Critical Care 2016, 20, 367. “Resting energy expenditure, calorie and protein consumption in critically ill patients: a retrospective cohort study”. Permission was obtained from the authors; © 2019 Copyright by Zusman et al.

As a result, predictive equations were introduced. In the last few years many studies comparing predictive equations to IC showed poor agreement results in various group of patients [25,26,27]. A recently published single-center retrospective study of 1440 intensive care patients found no significant correlation between the two [28]. In a large cohort retrospective study, Zusman et al. found a nonlinear association between administered calories and the 60-day mortality rate. As the number of calories administered reached 70% of resting energy expenditure (REE), a decrease in mortality was noted. As the caloric intake increased and reached >100%, the mortality rate increased as well, creating a U-shaped curve (see Figure 2) [29]. The use of indirect calorimetry limits the risk of overfeeding/underfeeding by determining a target based on measurements of energy expenditure. Therefore, various guidelines highly recommend using IC to determine energy requirements [17,18,22] Alternative methods to calculate energy expenditure (EE) have been proposed, including methods based on ventilated carbon dioxide (VCO2) measurements in mechanically ventilated patients [30]. Many mechanical ventilators can measure VCO2, which in turn can be used to calculate EE using Weir’s formula by assuming the respiratory quotient (RQ). This method remains controversial; *Rousing* et al. concluded that VCO2-based calorimetry is an accurate alternative to predictive equations with a 10% accuracy rate of 89% compared to IC [31], whereas Oshima et al. found end-expiratory VCO2 (EEVCO2) to be insufficiently accurate, with a 10% accuracy rate of 77% compared to IC [32]. It is important to note that EEVCO2 requires the use of a constant estimated RQ value; most studies use an RQ value of 0.85. Kagan et al. performed a retrospective observational study comparing IC-REE and VCO2-REE, finding that the level of agreement between the two REE measurements was highest when using an RQ value of 0.89 [33]. RQ is influenced by many factors, such as ventilation and acid–base balance, which are both highly unstable in critically ill patients [34], which is one of the reasons why this method is so controversial; however, although its drawbacks must be acknowledged, at this point it seems to be the best alternative to indirect calorimetry regarding energy expenditure estimation.

### 3.3. Venous Access Care and Infection Risks

Central venous catheters, both short-term and long-term, are associated with infectious complications, which, as mentioned above, is the main limitation of PN. Other than central line infections, PN increases the overall risk of infection, including pneumonia and intra-abdominal abscess [35]. A meta-analysis by Elke et al. on 18 RCTs including 3347 patients compared the clinical outcomes of enteral and parenteral nutrition in critical care patients. EN showed a significant reduction in rate of infection compered to PN, but this effect was only seen in a subgroup of patients where the PN group received a significantly higher caloric intake. Therefore, the positive effect of EN on the infection rate was attributed to the caloric intake gap between the two groups [8]. The same meta-analysis also found a significant publishing bias in trials demonstrating infection complications [8].

Global guidelines for the prevention of intravascular catheter-related infections [36,37,38,39] emphasize the importance of educational programs for healthcare workers and patients regarding infection protection and hand decontamination. A recently published RCT by Inchingolo et al. showed that educational programs with or without port protectors substantially reduced the rate of both central line-associated bloodstream infections and central venous colonization [40]. Choice of insertion site and proper insertion technique are other key issues regarding infection prevention. According to the Centre for Disease Control (CDC) guidelines for the prevention of intravascular catheter-related infections [39], the use of an upper extremity site for midline catheters is recommended, alongside daily inspection of the catheter site. For central catheters, the subclavian site is recommended to minimize infection risk. Results from the 3SITE study showed that the risk for catheter-related bloodstream infections or symptomatic vein thromboses in femoral sites was 3.1 times higher than in subclavian sites, and 2.1 times higher in jugular vein sites than in subclavian sites [41]. It was further recommended that an ultrasound be used to reduce the number of insertions attempts and, therefore, the chance of infection [37,38,39]. Other efforts are being investigated to further reduce infection complications, such as chlorhexidine-impregnated dressings [42,43,44], which is now also a part of recent global guidelines [37,38,39]. In an RCT by Wouters et al. comparing taurolidine locks to 0.9% saline locks, a significant reduction in catheter-related bloodstream infections were shown in patients with newly inserted central catheters. Therefore, taurolidine is a valid option for reducing infection rates [45] in home-based parenteral nutrition. If a line infection is suspected, the line should be removed and not replaced until the infection resolves.

### 3.4. Glucose Control

When PN was first introduced, it contained mainly glucose, either as a means to avoid protein degradation by suppressing amino acid oxidation or as a way to provide energy requirements in an era where lipid emulsions had severe side effects, such as chills, fever, nausea, vomiting, hypoxia, hypotension, and hemolytic anemia [46]. As a result, hyperglycemia became a serious concern, with multiple reports of its detrimental effects [47,48,49]. The development of safer and less inflammatory lipid emulsions decreased the carbohydrate contents of formulas and therefore the prevalence of hyperglycemia; however, hyperglycemia remains the most common complication of PN [34]. IV dextrose causes a more pronounced increase in blood glucose levels compared to the same amount received enterically due to IV dextrose bypassing the enteroinsular axis [34]. Van Der Berghe et al. concluded in an RCT that tight glycemic control of below 110 mg/dL reduced mortality and morbidity in critically ill surgical patients [50]. It was later shown by several studies, including the NICE-SUGER study, that intensive glucose control increased hypoglycemic events and mortality, and that a blood glucose target of 180 mg/dL resulted in better outcomes compared to lower targets [51,52,53]. According to current data, no specific glucose concentration range below the value of 180 mg/dL has further mortality benefits [54]. Variability in blood glucose concentrations is also known to be an important prognostic factor. Several studies demonstrated in heterogeneous populations of critically ill patients that increased glucose variability increased ICU and hospital mortality independently of the mean glucose concentration [55,56,57]. In recent years, the concept of time regarding glucose ranges proved to be of significant importance, with patients with normal blood glucose levels more than 80% of the time showing better outcomes [58,59,60]. Patients with or without diabetes may be affected differently by glucose concentrations [61]. Egi et al. concluded that nondiabetic patients tended to show significantly lower odds ratio of mortality with poor glycemic control when compared to diabetic patients [62]. Krinsley et al. did not find an association between glucose variability and increased mortality in a subpopulation of diabetic patients [63]. Another interesting element in glucose control is the presence of hyperlactatemia. Both glucose and lactate are found in higher concentrations in physiological stress; they are linked through the Cori cycle as part of glycolysis and gluconeogenesis. A few studies managed to show a strong correlation between these two compounds, demonstrating a significant increase in mortality when hyperglycemia and hyperlactatemia existed simultaneously. On the other hand, isolated hyperglycemia in the absence of hyperlactatemia was not shown to increase mortality [64,65,66]. Preventing hyperglycemia in PN is possible by starting nutrition slowly and constantly monitoring blood glucose levels. Total parenteral nutrition allows for better glucose control by controlling absolute blood glucose concentrations, decreasing hypoglycemic events, minimalizing glucose variability, or by increasing time in a normal range.

## 4. Development of Lipid Emulsion

As recommended in recent ESPEN guidelines and commonly practiced worldwide, lipid emulsions are an essential part of parenteral nutrition [18]. Throughout the years, significant improvements have been made regarding lipid emulsion compositions. Soybean oils, which are based on long-chain triglycerides (LCTs), were the first to be introduced, with medium-chain triglyceride (MCT)-based emulsions then being developed, followed by olive oil (N-9) and saturated lipid emulsions, and finally formulas containing fish oil. Current commercially available lipid emulsions contain different mixtures of oils and triglycerides [67]. Linoleic acid (LA) is an omega-6 (Ω-6) form of a fatty acid mainly found in soybean oil. During the past decade. many studies raised concerns about the safety of high concentrations of linoleic acid, mainly due to its proinflammatory and immunosuppressive properties [68,69]. *Heller* et al. found that the amount of Ω-6 infused into patients following gastrointestinal surgery was one of the two predictors of length of hospital stay, with the other being a delay in nutritional support [70]. Another well-known metabolic effect of LA is impaired synthesis of the omega-3 (Ω-3) polyunsaturated fatty acids eicosapentaenoic acid (EPA) and docosahexaenoic acid (DHA), which have central roles in inflammation, including in the immune response, coagulation, vasoactivation, and bone metabolism. It was also found to be essential in neuronal, behavioral, and visual development in infants [67,71]. Therefore, decreasing dietary consumption of Ω-6 fatty acids is recommended [18,72]. Olive oil-based formulas are a good alternative, with many in vitro and animal studies showing positive results [73]. A meta-analysis by *Dai* et al. found that olive oil-based emulsions showed nutritional benefits by increasing patients’ antioxidant levels and decreasing Ω-6 fatty acid levels. They also found the use of olive oil-based emulsion to be safe, with no significant differences in most liver enzyme levels [74]. As previously mentioned, fish-oil based emulsions are the most recent alternative, which are rich in long-chain Ω-3 fatty acids, with benefits including LA level reductions and Ω-3 fatty acids level increases. *de* Miranda Torrinhas et al. conducted an RCT in patients with gastrointestinal cancers, showing that a short-term, pre-operative infusion of a fish oil-based lipid emulsion was correlated with significant improvement in post-operative immune response; however, no significant clinical benefits were noted [75]. In another RCT, *Han* et al. compared parenteral nutrition with a mixture of soybean and MCTs with a fish-oil based emulsion in surgical ICU patients, showing a reduction in inflammatory mediators but only a nonsignificant reduction in liver dysfunction and infection rate [76]. Recently, Pradelli et al. published a meta-analysis on the use of Ω-3 fatty acid-enriched parenteral nutrition in hospitalized patients. They found that patients receiving Ω-3 fatty acids had lower infection rates and spent shorter periods in ICU, with a favorable but nonsignificant trend also being observed in mortality rates [77]. ESPEN guidelines conclude that fish oil-based lipid emulsions can be provided to patients receiving PN [18].

## 5. Improved Protein Intake

The development of amino acid solutions of parenteral nutrition has overcome many difficulties regarding formula instability and hyperammonemia. Older solutions did not contain tyrosine, glutamine, cysteine–cystine, or trace elements, which created a new problem [46]. Today, most standard parenteral solutions contain all amino acids in sufficient amounts, as recommended. Specialized solutions containing specific ratio of amino acid for certain clinical conditions are available [78]. Table 2 provides the protein contents of selected parenteral formulas. In recent years, observational studies showed benefits regarding high protein delivery to intensive care patients. *Nicolo* et al. found that achieving more than 80% of desired protein intake reduced mortality and shortened time to discharge [79]. Compher et al. showed similar results in intensive care patients with high nutrition risk (as represented by high NUTrition Risk in the critically ill score) [80]. Zusman et al. preformed a retrospective observational study on critically ill patients, showing a linear association between protein intake and decreased mortality, with a 1% decrease in mortality for every gram of ingested protein [29]. On the other hand, the EAT-ICU study showed less promising results; this RCT compared standard nutrition care in ICU patients to goal-directed nutritional care, relying on indirect calorimetry and nitrogen balance measurements. The control group received 1.2 g/kg/day of protein as a target and the goal-directed group received a minimum of 1.5 g/kg/day as a target. However, no effects on mortality, organ failure, or infection rate were found [81]. In a recent meta-analysis of a randomized control trial comparing high protein to low protein intake, no significant effects on mortality were noted [82]. In a post-hoc analysis of the EPaNIC trial, a high protein-to-glucose ratio showed no positive prognostic effects [8]. According to ESPEN guidelines in critically ill patients, 1.3 g/kgof protein should be delivered in acute illness [18]. However certain clinical conditions do require increased protein, for example, the target nitrogen supply in cancer patients is 1.2–2 g/kg [83], and burn patients require increased amino acid oxidation of up to 2 g/kg [84]. Timing is also a controversial issue in protein administration. *Bendavid* et al. concluded in a retrospective study that administrating protein during the early course of the disease (i.e., the first three days) was associated with better survival [85]. On the other hand, the PROTEINVENT retrospective study concluded that high protein intake during the first three to five days of admission to ICU was associated with increased mortality [86]. Until recently, suppling high levels of protein at such an early phase of the illness was only possible by using parenteral nutrition [87]. Newly developed enteral nutrition products containing high levels of protein open new possibilities, potentially providing the ability to reach protein targets while delivering less energy and volume [78].

## 6. Conclusions

Recent data regarding parenteral nutrition shows glucose control improvements and infection rate reductions, which, combined with the optimization of lipid emulsions and the frequent use of indirect calorimetry, make parenteral nutrition a valid option for nutritional support, both in acute and chronic artificially fed patients.

## Figures and Tables

**Table 1:** Studies comparing infection rates and clinical outcome in commercial bags vs. personalized compounding bags.

Study	Type of Study	Results
Turpin et al. 2011	Retrospective	Risk of BSI: 11.3% in commertial bags vs. 16.1% in personalized compounded bags, OR 1.56 (CI 1.37–1.79)
Turpin et al. 2012	Retrospective	Risk of BSI: 19.6% in commertial bags vs. 25.9% in personalized compounded bags, OR 1.54 (CI 1.39–1.69)
Pontes-Arruda et al. 2012	Prospective randomized	Incidence BSI:16.8% in commertial bags vs. 22.5% in personalized compounded bags. No significante difference in sepsis/septic shock incidence
Pontes-Arruda et al. 2012	Retrospective	Risk of BSI: 24.9% in commertial bags vs. 29.6% in personalized compounded bags, OR 1.29 (CI 1.06–1.59)
Turpin et al. 2014	Retrospective	Risk of BSI: HR 1.39 (CI 0.82–2.35) personalized compounded bags vs. commertial bags
		HR 1.85 (CI 1.17–2.94) commertial bags with ward addition vs. commertial bags alone
		HR 2.53 (CI 1.66–3.86) multibottle system vs.commertial bags
Liu et al. 2014	Retrospective	Rate of BSI: 19.6% in commertial bags vs. 25.9% in personalized compounded bags
		Rate of infection: 52.5% in commertial bags vs. 54.7% in personalized compounded bags
Magee et al. 2014	Retrospective	No significant difference between groups in infection rate

BSI- blood stream infection, OR-Odds ratio, HR- Hazard ratio, CI-Confidence interval.

**Table 2:** Protein and energy contents of common parenteral nutrition formulas.

Manufacturer	Product	Amino Acids (g/L)	Energy (kcal/L)
B Braun	Nutriflex	70	1054
Baxter	Clinimix	50	340
Baxter	Triomel 4	25	700
Baxter	Triomel 7	44	1140
Baxter	Triomel 9	56	1070
Fresenius	Smofkabiven	51	1116
